# Long-term variability in short-wavelength automated perimetry compared to standard perimetry in glaucoma patients and normal subjects

**DOI:** 10.4103/0974-620X.48419

**Published:** 2009

**Authors:** Chintan Malhotra

**Affiliations:** Department of Ophthalmology, Himalayan Institute of Medical Sciences, Jolly Grant, Dehradun, Uttarakhand, India

**Keywords:** Long-term variability, short-wavelength automated perimetry, white-on-white perimetry

## Abstract

**Aims:**

To compare long-term variability (LTV) in short-wavelength automated perimetry (SWAP) with that of standard white-on-white (W-W) perimetry, in primary open angle glaucoma (POAG) patients and normal subjects and to determine its clinical significance.

**Patients and Methods:**

The sample comprised 30 patients of stable POAG (group 1) and 20 age-matched normal controls (group2) who performed both SWAP and W-W perimetry thrice at monthly intervals. Long-term variability at each location in the visual field was calculated as the standard deviation of three threshold decibel values taken from the three visual fields performed. Mean LTV was then calculated for the entire field for each subgroup and compared.

**Results:**

Mean LTV in SWAP and W-W perimetry in glaucoma patients was 3.01 ± 0.74 dB and 2.73 ± 1.07dB respectively. In normal subjects mean LTV of SWAP and W-W perimetry was 2.69 ± 0.91 db and 1.97 ± 1.34 dB respectively. Intra group analysis revealed that mean LTV of SWAP was greater compared to W-W perimetry in normal subjects and glaucoma patients. In the latter, the difference did not reach statistical significance. Inter group analysis showed that mean LTV in both SWAP and W-W perimetry was greater in glaucoma patients than in normal subjects. Factors other than POAG that could influence LTV, including progression of cataract and change in pupil size due to use of pilocarpine were excluded. Mean examination time for SWAP per sitting was 7.65% longer than that for W-W perimetry.

**Conclusions:**

Higher mean LTV of SWAP as compared to W-W perimetry needs to be taken into consideration when evaluating serial visual fields for change. Use of rapid threshold algorithms e.g. SITA (Swedish interactive test algorithm) SWAP should be encouraged as these will reduce the patient fatigue and increase the reliability of the test.

## Introduction

Short-wavelength automated perimetry (SWAP) preferentially stimulates the short-wavelength sensitive pathway subserved by the large retinal ganglion cells while conventional white-on-white (W-W) perimetry examines the luminance pathway.[1] Various studies have demonstrated that SWAP can reflect early glaucomatous damage 3-5 years before the development of abnormality on standard W-W perimetry in patients with ocular hypertension.[2–4] Additionally SWAP defects are larger and the rate of progression is greater than that found for standard W-W automated perimetry in glaucoma patients.[4]

Any psychophysical test such as perimetry produces some variability in the results of subsequent examinations because the ability to detect a light stimulus of threshold value is not an all or none phenomenon. There is significant variation in the threshold for light detection at a given spot in normal eyes and this variability is particularly large in glaucomatous eyes.[5,6]

Previous studies have shown SWAP to have a greater short term variability (intra test variability) as well as a greater long-term variability(test retest variability)in normal subjects as well as glaucoma suspects and glaucoma patients as compared to standard W-W perimetry.[7–12] Thus interpretation of SWAP fields done over a period of time to assess progression of glaucomatous damage in a patient, may be more difficult as compared to the W-W visual fields, as SWAP will require greater change to be beyond variability limits. The present study was conducted to compare long-term variability (LTV) of SWAP with LTV of conventional W-W perimetry, in primary open angle glaucoma (POAG) patients and normal subjects belonging to Northern India and to determine its clinical significance, as literature search did not reveal such data in a North Indian population.

## Materials and Methods

Both conventional (W-W) automated perimetry and SWAP (blue-on-yellow perimetry) were performed on the Humphrey Field Analyzer 750 (Humphrey Instruments, SanLeandro, CA). The 24-2 full threshold programme with the standard 4-2 dB staircase strategy which utilizes double crossing of threshold for determination of the threshold value, was used. Oral informed consent was obtained from the subjects. No ethics review was felt to be required as the study did not involve any invasive or potentially harmful procedure on the study subjects.

Subjects were recruited in two groups. Group1 consisted of 30 patients diagnosed to have stable primary open angle glaucoma i.e. these patients showed no evidence of progressive visual field loss on three consecutive visits over the last 1 year, prior to enrollment in the study. One eye per patient was included in the study. All patients met the selection criteria shown in Table 1. Group 2 consisted of 20 randomly recruited age matched normal subjects (controls) who attended the out patient service of the institute for diseases other than glaucoma. For this group also one eye per patient was randomly selected. Table 2 shows the selection criteria for group 2.

**Table 1:** Selection criteria for Group 1 (Stable POAG* patients)

Inclusion criteria:
Age between 39 - 65 years. IOP** of 14 - 26 mm Hg in the study eye, with or without drugs, recorded on three separate visits (by Goldmann applanation tonometry), Evidence of stable[11] (non progressive) primary open angle glaucoma (POAG) which included an abnormal optic disc consistent with the diagnosis of glaucoma and repeatable glaucomatous 24-2 W-W visual field loss[11] at baseline. Best corrected visual acuity for distance better than or equal to 6/12
Exclusion criteria:
History of acute angle closure, congenital glaucoma, secondary glaucoma or ocular trauma. Prior intraocular surgery Significant refractive errors Evidence of any retinal disease likely to affect visual fields adversely Presence of clinically significant cataract in the study eye(lens opacity exceeding Lens Opacity Classification SystemIII standard photographs[12] nuclear colour 4, nuclear opalescence 4, cortical cataract 3, or posterior subcapsular cataract 2) Congenital colour vision defects Patients with advanced visual field loss (mean deviation worse than -10dB) and field loss threatening fixation. Optic nerve disorders not attributable to glaucoma

* POAG -primary open angle glaucoma

** IOP-intraocular pressure

**Table 2:** Selection criteria for group 2 (Normal subjects)

Inclusion criteria:
IOP* of less than 21 mmHg in the study eye on 3 separate visits by Goldmann applanation tonometry Normal optic nerve head appearance. Normal white-on-white perimetry 24-2 visual fields. Visual acuity of 6/12 or better.
Exclusion criteria:
Congenital colour vision defects. Clinically significant cataract. (lens opacity exceeding Lens Opacity Classification SystemIII standard photographs[12] nuclear colour 4, nuclear opalescence 4, cortical cataract 3, or posterior subcapsular cataract 2) Prior intraocular surgery Significant refractive error Evidence of any retinal disease likely to affect visual fields adversely History of ocular trauma. Family history of glaucoma.

* IOP-Intraocular pressure

Subjects in both groups were enrolled after performing preliminary visual fields (one W-W and one SWAP visual field) in the study eye, to determine reliability and to reduce the likelihood of a residual learning curve effect on long-term variability. The preliminary visual fields were used only to select patients for the study and were not used for data analysis. Both standard W-W and SWAP visual fields were performed on the same visit separated by an interval of 10 minutes. The order in which the two tests were performed was randomized between patients and visits. Following the preliminary fields, three field examinations were done at one, two and three months respectively.

**Figure 1:**
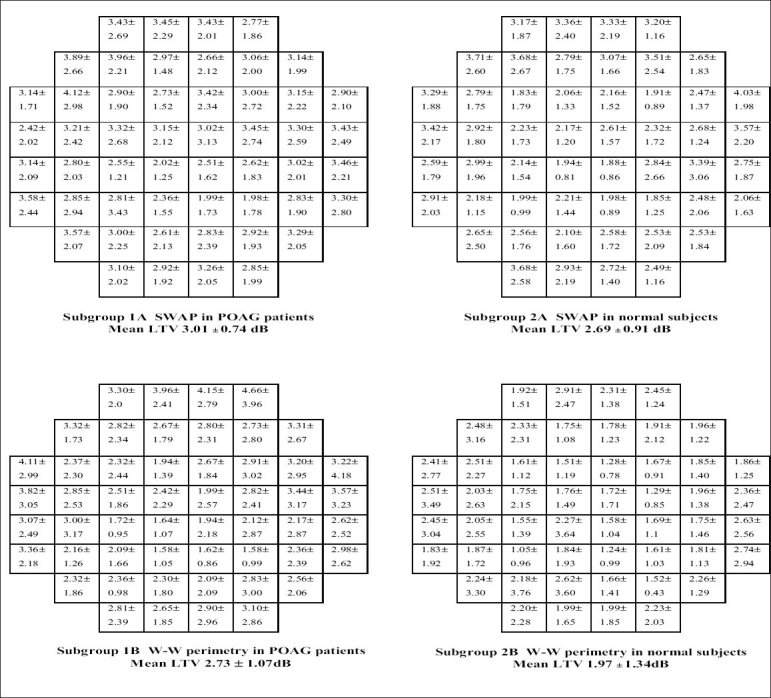
Average long-term variability (LTV) of all patients in a subgroup, at each of the 52 locations in the 24-2visual field (excluding the two points immediately above and below the blind spot). Each small square represents a test location in the field of vision.

Data was tabulated and analyzed in 4 subgroups, as shown in Table 3. LTV at a point was calculated as the standard deviation of the three threshold dB values at that particular point in the visual field,[11] taken from the three visual fields performed. The LTV was calculated separately for each of the 52 visual field locations (excluding the two points immediately above and below the blind spot) for every patient in all the 4 subgroups. Then for each point the LTV of all the patients in a particular subgroup was averaged ± SD [Figure 1]. The mean LTV for the entire 24-2 visual field was then calculated as the average of LTV ± SD of all the 52 points in the field for each of the four subgroups. LTV of each subgroup was compared by the ‘t’ and paired ‘t’ test, as appropriate, for statistical significance [Table 4].

**Table 3:** Categories of subgroups

Group1: Stable POAG* patients
1A SWAP** in POAG patients 1B W-W*** perimetry in POAG patients
Group2: Normal subjects
2A SWAP in normal subjects 2B W-W perimetry in normal subjects

* POAG-primary open angle glaucoma,

** SWAP-Short wavelength automated Perimetry,

*** W-W-white on white

## Results

Mean age of glaucoma patients (group 1) was 53.93 years (range 40-68 years), while that of normal subjects (group 2) was 50.3 years (range 40-65 years). The difference in age between the two groups was not statistically significant (*P* > 0.1). Mean LTV for each subgroup is shown in Figure 1. Table 4 shows the statistical significance of the difference in mean LTV of the 4 subgroups when these were compared with each other. Mean LTV of SWAP in glaucoma patients (subgroup 1A) was greater than mean LTV of W-W perimetry in glaucoma patients (subgroup 1B) but the difference was not statistically significant [p > 0.1]. Mean LTV of SWAP in normal subjects (subgroup 2A) was significantly greater than LTV of W-W perimetry in normals (subgroup 2B) [*P* < 0.01]. Mean LTV of SWAP in glaucoma patients (subgroup 1A) was greater than mean LTV of SWAP in normal subjects (subgroup 2A) and the difference again was statistically significant [*P* < 0.5] Mean LTV of W-W perimetry in glaucoma patients (subgroup 1B) was greater than mean LTV of W-W perimetry in normal subjects (subgroup 2B) and the difference was statistically significant [*P*< 0.05].

**Table 4:** Comparison of mean LTV of subgroups

*Comparison of mean LTV of subgroups*	*P value*	*Statistical significance*
1A>1B	>0.1	NS
2A>2B	<0.01	S
1A>2A	<0.05	S
1B>2B	<0.05	S

LTV-long-term variability, *P* value-Probability value. NS-not significant. S-significant. >-greater than, <-less than (subgroups were compared by ‘t’ and paired ‘t’ test)

**Figure 2:**
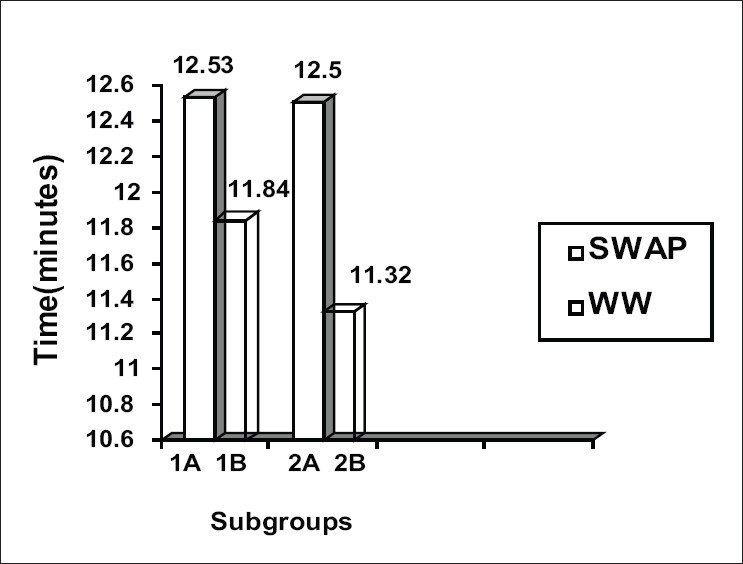
Mean examination time i.e examination time per patient per sitting in each of the 4 subgroups. Glaucoma patients took 5.83% longer to do SWAP while normal subjects took 10.42% longer to do SWAP as compared to white-on-white perimetry. (Subgroup 1A - SWAP in glaucoma patients, 1B-white-on-white perimetry in glaucoma patients, 2A- SWAP in normal subjects, 2B- white-on-white perimetry in normal subjects).

Mean examination time (i.e. examination time per patient per sitting) was determined and is shown in Figure 2. Glaucoma patients took 5.83% longer time to do SWAP (12.53 min.) as compared to W-W perimetry (11.84 min) [*P* < 0.05]. Normal subjects took 10.42% longer time to do SWAP (12.50 min) than to do W-W perimetry (11.32min)[*P* < 0.01]. Overall mean examination time for SWAP (12.52 min.) was 7.65% longer than the mean time for W-W perimetry (11.63 min), when both groups were considered together.

## Discussion

True glaucomatous progression, learning effect and poor reliability parameters can all act as possible confounders for determination of LTV component of inter-visit fluctuations. To isolate these confounders, only patients with stable open angle glaucoma were included in group1 and subjects in both groups 1 and 2 were required to perform preliminary visual fields, which were however not considered for data analysis. Also as SWAP visual field is more adversely influenced by ocular media absorption than the W-W visual field,[1] patients with significant cataract,[13] were excluded to lessen the magnitude of reduction in sensitivity due to age related lenticular changes.

Long-term variability of W-W perimetry in glaucoma patients (2.73 ±1.07dB) was significantly [*P* < 0.05] greater than LTV of W-W perimetry in normal subjects (1.97±1.34dB) in the current study. LTV of SWAP in glaucoma patients (3.01 ± 0.74 dB) was also found to be significantly [*P* < 0.05] higher than LTV of SWAP in normal subjects (2.69± 0.91 dB). As other factors which could adversely affect the fields in glaucoma patients i.e. changes in pupil size due to use of pilocarpine or progression of cataract were excluded, the greater LTV of both the techniques in glaucoma patients suggests that these patients may be performing perimetry poorly as compared to normals because of the basic underlying pathology of the glaucomatous process which predisposes to increased variability.

In the present study LTV of SWAP in normal subjects (2.69 ± 0.91dB) was greater than LTV of W-W perimetry in the same group (1.97 ± 1.34dB) and the difference was statistically significant [*P*< 0.01]. This finding is consistent with a previous study by Kwon *et al*,[7] (on young normal subjects without visual field defects) who also found that the long-term fluctuation for blue-on-yellow perimetry (4.07±3.07dB^2^) was significantly greater than that for W-W perimetry (1.97±0.99dB^2^) [*P*< 0.001]. Similar results have also been reported in another study.[9] Previous studies have attributed this difference to the relative inexperience of the subjects with blue-on- yellow perimetry[9] or a longer duration of the learning effect for blue-on-yellow perimetry as compared to W-W perimetry.[14] Kwon *et al*,[7] have speculated that at least part of the reason for the greater long-term fluctuation of SWAP is the decreased sampling of retinal neuronal elements in SWAP as compared to W-W perimetry. The blue and yellow sensitive channels comprise about 6% of the total number of retinal ganglion cells[15] and thus SWAP relies on only a fraction of retinal neurons for its psychophysical response. W-W perimetry, on the other hand, recruits many more neural elements including short-wavelength, middle wavelength and long wavelength sensitive channels for its response. Possibly the response generated from a small fraction of retinal neuronal elements shows greater variability or “noise”. Interestingly, the same decreased sampling in SWAP may facilitate detection of early retinal ganglion cell loss in glaucoma when compared to W-W perimetry. In this study mean examination time for SWAP in normal subjects was 10.42% longer than the time taken for W-W perimetry. Other studies have also reported an increased time taken to perform full threshold SWAP as compared to a full threshold W-W perimetry.[16] Thus in addition to the above factors the fatigue caused by the increased examination time for SWAP may cause subjects to perform the test poorly, accounting for the increased LTV of SWAP as compared to W-W perimetry.

Long-term variability of SWAP in glaucoma patients (3.01 ± 0.74dB) was also found to be greater than LTV of W-W perimetry in glaucoma patients (2.73 ± 1.07dB) in the current study, but the difference did not reach statistical significance (*P* > 0.1). Blumenthal *et al*,[11] also found the LTV of SWAP in glaucoma patients (2.92 ± 2.03dB) to be greater than LTV of W-W perimetry in glaucoma patients (2.37 ± 2.03dB) by an average of 0.55dB and the difference in their study was statistically significant (*P* . 0.0001). This discrepancy may possibly be explained by the different statistical methods used for analysis in the two studies. As in normal subjects, the greater LTV of SWAP in glaucoma patients as compared to W-W perimetry may again be explained by the lesser number of neurons subserving the blue-on- yellow pathway well as the longer time taken to perform SWAP (glaucoma patients took 5.83% longer for SWAP than W-W perimetry in our study). Algorithms like SITA SWAP which take considerably less time than the standard full threshold SWAP while retaining the same diagnostic sensitivity,[17,18] may thus prove to be extremely useful in eliminating this fatigue factor.

One drawback of this study was that global summaries of LTV were considered, and LTV was not compared at different visual field locations. Previous studies have shown that farther away from fixation a tested point lies, the larger the LTV.[19] Also sometimes these variabilities could be signaling of or preceding actual field defect in the susceptible areas of the field.

The results of this study conducted in a subset of North Indian population matched those reported in literature from Caucasian and other populations. However LTV differences of less than 1dB between standard W-W visual fields and SWAP (as seen in the study by Blumenthal *et al* and in this study) may not be of clinical significance in following up individual patients[11] and hence SWAP will continue to complement W-W perimetry in the diagnosis and follow of cases of early glaucomatous damage.
